# Predetermination of Sex

**Published:** 1914-06

**Authors:** Israel Bram

**Affiliations:** Philadelphia, Instructor in Physical Diagnosis, Medico-Chirurgical College


### Predetermination of Sex.

BY ISRAEL BRAM, M. D., PHILADELPHIA, INSTRUCTOR IN PHYSICAL DIAGNOSIS, MEDICO-CHIRURGICAL COLLEGE.

Numerous theories have been advanced from time to time to solve the mystery of the determination of sex in the unborn being. The degree of potency of one or the other parent, hereditary influences, season of the year at the time of conception, the intellectual status of one or both parents, the age of the parents, the state of mind of the parents at the time of the conjugal embrace, the degree of amativeness during that time, emotional stress, the presence or absence of diseases of one or more organs (particularly the adrenal glands) in the maternal economy, the quality or quantity of the food of the mother, and numerous other theories have been advanced as factors in the determination of whether the resulting infant will be male or female.

The results of my investigations are the outcome of the utilization of a combination and modification of the following two most plausible theories:

1. Sex in offspring is the outcome of a contest between the spermatazoon and the ovum (Weill). If the spermatazoon is successful, the outcome is a male; if the ovum, the offspring is a female. This contest varies in one direction or the other by the influence of food. An excess of nourishment in the mother may decide in favor of a female, and a deficiency in the production of a male. Consequently, generous quantities of food with an excess of albuminous nitrogenous substances may decide at conception in favor of a baby girl, and a scanty diet with a very low proteid allowance in favor of a baby boy. In support of this theory it is advanced that in times of financial panic, war, etc., when a plentiful quantity of rich, nitrogenous food is not obtainable, male infants are in preponderance.

2. Sex is determined by the activity of the adrenal glands of the prospective mother (R. Robinson). In support of this theory it is asserted that in a large majority of women who present evident signs of adrenal insufficiency (pigmentation of the skin, marked gastric disturbances, headaches, etc.), the offspring are female. The use of the extract of suprarenal gland or something which would stimulate the adrenal glands to increased physiological function, seems to be indicated in this class of cases.

Among my obstetrical patients during the past two years, there were a considerable number who manifested their eagerness to give birth to male children. Some were primiparae, while others had already one or more female children. In an effort to test the value of my views concerning the predetermination of sex, I selected thirty of these patients, twenty of whom had reported to me during the first two months of pregnancy, explaining the harmlessness of the treatment that I was about to offer and the possibility of having their desires fulfilled. Of the ten who had reported to me *after* the second month of pregnancy, six gave birth to male children. The remaining twenty who had reported *during* the first two months all gave birth to *male* children. The ages of these twenty women vary from nineteen to thirty-six years; and their obstetrical records were as follows:

Four were primiparae.

Five had each previously given birth to three girls.

Five had each previously given birth to two girls.

One had previously given birth to two girls and one boy.

One had previously given birth to two girls and two boys.

One had previously given birth to five girls and one boy.

Two had previously given birth to three girls and one boy.

One had previously given birth to four girls.

In the last case the outcome was twin boys.

These results strongly indicate that the experiment employed was highly successful, and th[illegible] we probably have at our disposal a means of controlling at will [illegible]e sex of the unborn infant if the prospective mother present herself early enough to the obstetrician, i. e., on the first manifestations of pregnancy.

According to theories No. 1 and 2, if a boy is desired, the treatment is essentially dietary and medicinal, and is persisted in until the fourth or fifth month of pregnancy, when it may be discontinued. Albuminous, nitrogenous substances, such as eggs, meat, fish, cheese, etc., were forbidden; the diet consisting essentially of such substances as cereals, fruits, potatoes, milk, buttermilk, and butter. Plenty of water was insisted upon to keep the kidneys active. In addition the patient was given after each meal a capsule consisting of two grains of the extract of suprarenal gland, combined with four grains of lecithin. The reason for administering the extract of suprarenal gland has already been outlined in theory No. 2.

About three years ago, I had under observation a typical case of Addison's disease, in which, though the extract of suprarenal gland alone resulted in little or no benefit, the symptoms were markedly ameliorated for many months by the addition of lecithin. I have also obtained excellent results in marked cases of chloasma by the administration of the extract of suprarenal gland with lecithin. It is a well known fact that the combination of the iodide of potassium with the extract of thyroid gland is a desirable one, as the former substance seems to enhance the action of the latter to considerable degree. The combination of lecithin with the extract of the suprarenal glands seems likewise to increase the action of the latter in cases where there is adrenal insufficiency. It is highly possible, also, that lecithin stimulates the normal adrenal gland to increased function.

To summarize, it is safe to say that we probably have at our disposal a definite means for predetermination of the male sex; that the essentials in the treatment are: 1. It should be instituted immediately after the woman has missed her period; 2. foods rich in albuminous nitrogenous elements should be interdicted; 3. the administration of the extract of suprarenal gland combined with lecithin is required.—*New York Medical Journal.*

